# Chemical Constituents and Pharmacological Activities of Garlic (*Allium sativum* L.): A Review

**DOI:** 10.3390/nu12030872

**Published:** 2020-03-24

**Authors:** Gaber El-Saber Batiha, Amany Magdy Beshbishy, Lamiaa G. Wasef, Yaser H. A. Elewa, Ahmed A. Al-Sagan, Mohamed E. Abd El-Hack, Ayman E. Taha, Yasmina M. Abd-Elhakim, Hari Prasad Devkota

**Affiliations:** 1 National Research Center for Protozoan Diseases, Obihiro University of Agriculture and Veterinary Medicine, Nishi 2-13, Inada-cho, Obihiro, Hokkaido 080-8555, Japan; amanimagdi2008@gmail.com; 2 Department of Pharmacology and Therapeutics, Faculty of Veterinary Medicine, Damanhour University, Damanhour 22511, AlBeheira, Egypt; lamiaawasef@vetmed.dmu.edu.eg; 3 Department of Histology and Cytology, Faculty of Veterinary Medicine, Zagazig University, Zagazig 44511, Egypt; y-elewa@vetmed.hokudai.ac.jp; 4 Laboratory of Anatomy, Department of Biomedical Sciences, Graduate School of Veterinary Medicine, Hokkaido University, Sapporo, Hokkaido 060-0818, Japan; 5 King Abdulaziz City for Science and Technology, P.O. Box 6086, Riyadh 11442, Saudi Arabia; abdeen@kacst.edu.sa; 6 Department of Poultry, Faculty of Agriculture, Zagazig University, Zagazig 44511, Egypt; dr.mohamed.e.abdalhaq@gmail.com; 7 Department of Animal Husbandry and Animal Wealth Development, Faculty of Veterinary Medicine, Alexandria University, Edfina 22578, Egypt; Ayman.Taha@alexu.edu.eg; 8 Department of Forensic Medicine and Toxicology, Faculty of Veterinary Medicine, Zagazig University, Zagazig 44511, Egypt; yasmina.forensic@yahoo.com; 9 Graduate School of Pharmaceutical Sciences, Kumamoto University, 5-1 Oe-Honmachi, Chuo-ku, Kumamoto City, Kumamoto, 862-0973, Japan; devkotah@kumamoto-u.ac.jp

**Keywords:** Garlic, *Allium sativum*, pharmacological activities, phytoconstituents, allicin, pharmacokinetics

## Abstract

Medicinal plants have been used from ancient times for human healthcare as in the form of traditional medicines, spices, and other food components. Garlic (*Allium sativum* L.) is an aromatic herbaceous plant that is consumed worldwide as food and traditional remedy for various diseases. It has been reported to possess several biological properties including anticarcinogenic, antioxidant, antidiabetic, renoprotective, anti-atherosclerotic, antibacterial, antifungal, and antihypertensive activities in traditional medicines. *A. sativum* is rich in several sulfur-containing phytoconstituents such as alliin, allicin, ajoenes, vinyldithiins, and flavonoids such as quercetin. Extracts and isolated compounds of *A. sativum* have been evaluated for various biological activities including antibacterial, antiviral, antifungal, antiprotozoal, antioxidant, anti-inflammatory, and anticancer activities among others. This review examines the phytochemical composition, pharmacokinetics, and pharmacological activities of *A. sativum* extracts as well as its main active constituent, allicin.

## 1. Introduction

Medicinal plants have been a good source of new pharmacologically active molecules. For example, natural products could be a potential alternative for controlling the pathogen associated with diseases [1,2,3,4,5]. Recently, antibiotics and most drugs on the market have shown unwanted symptoms and the emergence of resistant pathogenic microorganisms, toxic effects related to these drugs, and withdrawal issues restricting their use in many countries [6], therefore, much attention has been paid to the herbal extracts and pharmacologically active molecules extracted from different plant species that are used previously in the traditional medicine [7,8,9,10,11]. Many plant species have been reported to exert pharmacological properties due to their phytoconstituents such as glycosides, alkaloids, saponins, steroids, flavonoids, tannins, and terpenoids (e.g., monoterpenes, diterpenes, and sesquiterpenes). Nowadays, eighty percent of the world’s populations depend on traditional medicines as an essential source of their primary health care [12,13]. Medicinal plant extracts and their constituents also possess various biological activities including virucidal, bactericidal, fungicidal, anti-inflammatory, analgesic, sedative, spasmolytic, and local anesthetic activities among others [14,15]. 

Garlic (*Allium sativum* L.; Family: Amaryllidaceae) is an aromatic herbaceous annual spice and one of the oldest authenticated and most important herbs that have been used from ancient times as traditional medicine [16,17]. It is considered the second broadly used *Allium* species with onion (*Allium cepa* L.), which is used as a remedy against several common diseases such are cold, influenza, snake bites, and hypertension [18]. *Allium* species and their active components are reported to reduce the risk of diabetes and cardiovascular diseases, protect against infections by activating the immune system and have antimicrobial, antifungal, anti-aging as well as anti-cancer properties which confirmed by epidemiological data from human clinical studies [19]. Garlic has been used for cooking purposes as a spice that can flavor foods during the cooking process. As well, it possesses therapeutic purposes including the treatment of lung disorders, whooping cough, stomach disorders, cold, earache, and assists in preventing cardiovascular disease [17]. While aged garlic extract (AGE), prepared from aged garlic is a folk herbal remedy that has been shown to enhance the immune system and thus inhibit cancer and heart disorders. Raw garlic and its transformed products have been reported to contain various sulfur compounds that have been included in several types of preparations [20]. Moreover, quercetin, the major flavonoid isolated from garlic, was found to interact with some medications such as vitamin E and C [21] and modify the in vitro as well as the in vivo transferases and cytochrome P450 isozymes activity, however, the in vivo studies revealed that garlic oil and its three allyl sulfide components increase the CYP3A1, 2B1, and 1A1 expression in the hepatic detoxification system [22]. 

Allicin [S-(2-propenyl)-2-propene-1-sulfinothioate], the most biologically active sulfur-containing compound of garlic, is responsible for its smell and taste [23,24]. Alliin (S-allyl-L-cysteine sulfoxide) is the main precursor of allicin, which represents about 70% of total thiosulfinates existing in the crushed cloves [25]. Allyl mercaptan is the odorant molecule responsible for the garlic breath and results from the interaction of allicin or diallyl disulfide with cysteine in the presence of S-ally-mercapto cysteine [25,26]. Allicin is a lipid-soluble sulfur compound, which can be easily damaged by cooking and has the ability to provoke intolerance, allergic reactions, and gastrointestinal disorders [27,28,29]. Various pharmacological activities have also been reported for the extracts and isolated compounds from garlic. The main aim of this article is to critically review the available scientific information about the traditional uses, chemical constituents, pharmacokinetics, and pharmacological activities of garlic. 

## 2. Chemical Constituents of Garlic

Bulbs of *A. sativum* are reported to contain hundreds of phytochemicals including sulfur-containing compounds (Table 1) such as ajoenes (*E*-ajoene, *Z*-ajoene), thiosulfinates (allicin), vinyldithiins (2-vinyl-(4H) -1,3-dithiin, 3-vinyl-(4H)-1,2-dithiin), sulfides (diallyl disulfide (DADS), diallyl trisulfide (DATS)) and others that accounted 82% of the overall garlic sulfur content [30]. Alliin, the main cysteine sulfoxide is transformed to allicin by allinase enzyme after cutting off the garlic and breaking down the parenchyma [31]. S-propyl-cysteine-sulfoxide (PCSO), allicin and S-methyl cysteine-sulfoxide (MCSO) are the main odoriferous molecules of freshly milled garlic homogenates [31]. PCSO can produce more than fifty metabolites depend on water content and temperature as well as allinase enzyme that can act on the mixture of MCSO, PCSO, and alliin to produce other molecules, such as allyl methane thiosulfinates, methyl methanethiosulfonate, and further corresponding thiosulfinates (R-S-S-R′), by which R and R′ are allyl, propyl, and methyls groups [31]. 

S-alk(en)yl-l-cysteine sulfoxides are the secondary metabolites obtained from cysteine which accumulate in the plants of *Allium* genus [32]. Garlic formulations consist of several organosulfur compounds, N-acetylcysteine (NAC), S-allyl-cysteine (SAC) [33], and S-ally-mercapto cysteine (SAMC), which are derived from alliin [34]. Notably, SAC has antioxidant, anti-inflammation, regulated redox, pro energetic, antiapoptotic, and signaling capacities [32,35], while SAMC shows an anticancer activity through preventing the cancer cells multiplication [36]. 

Allicin (allyl thiosulfinate), is a sulfenic acid thioester and its pharmacological effect is attributed to its antioxidant activity as well as its interaction with thiol-containing proteins [37]. In the allicin biosynthesis, cysteine is transformed to alliin that is hydrolyzed by the allinase enzyme [38]. This enzyme composed of pyridoxal phosphate (PLP) which splits alliin and produces ammonium, pyruvate, and allyl sulfenic acid that are highly reactive and unstable at room temperature, where two molecules were combined to form allicin [37,39].

## 3. Pharmacokinetics and Stability of Garlic Components

De Rooij et al. [40] reported the existence of N-acetyl-S-allyl cysteine (NASAC) in human urine after garlic ingestion that is created by converting SAC into the N-acetylated metabolite by N-acetyl transferase enzyme. Previous reports revealed the existence of diallyl disulfide (DADS), allyl methyl sulfide (AMS), diallyl sulfide (DAS), allyl methyl disulfide (AMDS), dimethyl sulfide, acetone and diallyl trisulfide (DATS) in tested participants breath after administration of 38 g of raw garlic. It was reported that DADS, DAS, DATS, and AMDS achieved the maximum concentrations within 2 to 3 h. Freeman and Kodera [41] examined allicin stability in simulated gastric fluid (SGF), blood, stimulated intestinal fluid (SIF) and various solvents such as; methanol, water, ethyl acetate at pH 1.2 and 7.5 and they observed that allicin affected the SGF and SIF at pH 1.2 and 7.5, respectively. These results suggest that allicin degraded at room temperature and was more stable in methanol than in ethyl acetate. Furthermore, about 90% of the allicin stayed in the SIF (pH 7.5) and SGF (pH 1.2) after incubation at 37 °C for 5 h, while only a small amount could be detected after 5 min in the blood. About 62% and 80% of allicin remained one day after *Allium* administration without increasing the concentration of allicin decomposition products such as DADS [40]. The pharmacokinetic examination in rats using 35 S-labeled alliin, vinyl dithiins, and allicin, revealed that the alliin peak time (T_max_) was lower than 10 min and was eliminated after 6 h from the blood, whereas the allicin peak time (T_max_) was 30–60 min and the mean total fecal and urinary excretion was 85.5% after 72 h.

Allicin, a bright yellow oily liquid that possesses a distinctive garlic odor and it is very unstable, therefore it can be easily decomposed even at room temperature [42]. Previous studies reported that allicin can easily degrade under the influence of temperature to form ajoenes ((*E*)- and (*Z*)-4, 5, 9-trithiadodeca-1, 6, 11-triene-9-oxides) and vinyldithiins which are more stable than allicin [43]. These degradation products are commonly isolated from oil, aqueous and chloroform garlic extracts and are present as (*E*) and (*Z*) isomers, where (*E*)-ajoene is usually found in double amounts [44]. 

## 4. Pharmacological Activities of Garlic and Its Related Compounds

### 4.1. Traditional Uses of Garlic

Garlic is one of the most important bulb vegetables that has a pungent flavor and widely used all over the world as a spice and flavoring agent. The organosulfur compounds like allicin and DADS are the main compounds responsible for its pungency effects and spicy aroma. Garlic is well-known to be used in food preparation, especially dried foods for storage and some types of soup and it can be utilized in both fresh and dehydrated states [45]. Traditionally, garlic and its related compounds have been stated to have several biological activities including anticarcinogenic, antioxidant [46], antidiabetic, renoprotective, anti-atherosclerotic, antibacterial, antifungal [47], and antihypertensive activities [17]. Moreover, garlic has been used in traditional medicine to treat indigestion, respiratory and urinary tract infections and cardiac disorders and it showed carminative, antipyretic, sedative, aphrodisiac, and diuretic effects [32]. 

### 4.2. Activities Related to Infectious Diseases

#### 4.2.1. Antibacterial Activity

The antimicrobial activity of garlic is attributed to allicin activity that was reported toward a wide variety of microorganisms including antibiotic-resistant, Gram-positive and Gram-negative bacteria such as *Shigella, Escherichia coli* [48], *Staphylococcus aureus*, *Pseudomonas aeruginosa* [44], *Streptococcus mutans*, *S. faecalis*, *S. pyogenes*, *Salmonella enterica, Klebsiella aerogenes* [49], *Vibrio*, *Mycobacteria*, *Proteus vulgaris,* and *Enterococcus faecalis* [50]. Various garlic extracts (aqueous, chloroform, methanolic, and ethanolic extracts) were reported to inhibit the growth of several pathogenic bacteria with varying degrees of susceptibility. For instance, a study revealed that ethanolic garlic extract showed higher inhibitory effect against *E. coli* and *Sal. typhi* than the aqueous extract that showed little or no inhibition effect [51]. Meriga et al. [52] reported that aqueous garlic extract showed antibacterial activity toward Gram-negative (*Kl. pneumoniae* and *E. coli*) as well as Gram-positive (e.g., *Bacillus subtilis* and *S. aureus*) strains, whereas methanolic garlic extract showed antimicrobial activity against all tested strains except *S. aureus*. However, hexane, ethyl acetate, and chloroform extracts did not show any antibacterial effect. Moreover, garlic extracts prevented the growth of enterotoxigenic *E. coli* strains and other pathogenic intestinal bacteria, which are the main cause of diarrhea in humans and animals. Besides the antibacterial activity of garlic, it was reported to prevent the toxins produced by bacterial infection [53]. Allicin also showed effectiveness toward methicillin-resistant *S. aureus* (MRSA) [50]. Allicin’s antimicrobial activity is due to its chemical interaction with enzymes containing thiol e.g., thioredoxin reductase, RNA polymerase, and alcohol dehydrogenase [54] by oxidizing protein cysteine or glutathione residues under physiological conditions. Allicin is a dose-related biocide that can influence essential metabolism of cysteine proteinase, and thus, kill all eukaryotic cells due to the presence of thiol groups in all living cells. 

#### 4.2.2. Antifungal Activity

Garlic extracts showed a broad spectrum fungicidal effect against a wide range of fungi including *Candida, Torulopsis, Trichophyton, Cryptococcus, Aspergillus*, *Trichosporon,* and *Rhodotorula* species. Recently, garlic extract was found to inhibit the *Meyerozyma guilliermondii* and *Rhodotorula mucilaginosa* germination and growth [55]. Another study reported the antifungal activity of various *A. sativum* extracts namely aqueous, ethanolic, methanolic, and petroleum ether against human pathogenic fungi such are *Trichophyton verrucosum*, *T. mentagrophytes*, *T. rubrum*, *Botrytis cinerea*, *Candida* species, *Epidermophyton floccosum*, *Aspergillus niger*, *A. flavus*, *Rhizopus stolonifera, Microsporum gypseum, M. audouinii*, *Alternaria alternate, Neofabraea alba,* and *Penicillium expansum* [56]. The garlic extract acted by affecting the fungal cell wall and causing irreversible ultrastructural changes in the fungal cells, which lead to loss of structural integrity and affected the germination ability. These changes in the cytoplasmic content lead to nucleus and cell organelles damage that ultimately leads to cell death. Moreover, allicin and garlic oil showed potent antifungal effects against *Candida albicans, Ascosphaera apisin,* and *A. niger* [44] and they acted by penetrating the cellular membrane as well as organelles membranes like the mitochondria and leading to organelles destruction and cell death [57]. DADS and DATS separated from garlic essential oil showed antifungal activity against a number of fungi (*C. albicans*, *C. tropicalis*, and *Blastoschizomyces capitatus*). In addition to that, saponins extracted from *A. sativum* exhibited antifungal activity against *Botrytis cinerea* and *Trichoderma harzianum* [58].

#### 4.2.3. Anti-Protozoal Activity

Various studies reported the anti-protozoal activity of garlic extracts and its phytochemicals against several protozoan parasites. For instance, an in vitro study revealed that the aqueous, ethanolic, and dichloromethane *A. sativum* extracts exhibited anthelmintic activity against *Haemonchus contortus* and the ethanolic extract was the most effective one, while aqueous garlic extract showed potent activity against *Trichuris muris* and *Angiostrongylus cantonensis* [58]. Garlic was also examined in vivo and in vitro against *Taenia taeniaeformis*, *Hymenolepis microstoma*, *H. diminuta*, *Echinostoma caproni,* and *Fasciola hepatica* [59]. Abdel-Hafeez et al. [60] showed that garlic extract inhibited the growth of *Blastocystis* spp. in vivo and this activity attributed to that garlic extracts contains several phytochemicals e.g., thiosulfinates are one of the bioactive compounds that possess antibacterial activity that is related to thiol enzymes inhibition which presents in several microorganisms. Allicin also acts by preventing the parasite’s RNA as well as DNA and protein synthesis. Moreover, allicin and DATS, phytochemicals isolated from garlic extract, showed antiparasitic activity against *Entamoeba histolytica, Plasmodium falciparum, Babesia, Theleria, Trypanosoma brucei,* and *Giardia lamblia* [58]. Ajoene also exhibited antiparasitic activity by inhibiting the human glutathione reductase and *T. cruzi* trypanothione reductase [61]. Hazaa et al. [62] reported the activity of garlic oil toward broad-spectrum microorganisms such are *Cochlospermum planchonii, Plasmodium, Giardia*, *Leishmania*, and *Trypanosoma.*

#### 4.2.4. Antiviral Activity

The antiviral activity of garlic extracts has been evaluated against influenza B, human rhinovirus type 2, human cytomegalovirus (HCMV), Parainfluenza virus type 3, herpes simplex type 1 and 2, vaccinia virus, and vesicular stomatitis virus [63]. Interestingly, in vivo experiment exhibited the antiviral activity of garlic extract and they reported that garlic showed protective activity against influenza viruses by improving the production of neutralizing antibodies when given to mice and this activity was based on the presence of several phytochemicals namely, ajoene, allicin, allyl methyl thiosulfinate, and methyl allyl thiosulfinate [64]. Allicin acts by preventing several thiol enzymes, while ajoene’s antiviral activity was due to the prevention of adhesive interaction and fusion of leukocytes. Moreover, DATS was effective against the HCMV replication and viral immediate-early gene expression and it acts by enhancing natural killer-cell (NK-cell) activity that destroys virus-infected cells [58].

### 4.3. Antioxidant and Anti-inflammatory Activities 

#### 4.3.1. Antioxidant Activity

Asdaq and Inamdar [33] reported that the frequent garlic intake promotes internal antioxidant activities and reduces oxidative adverse effects either by increasing the endogenous antioxidant synthesis or reducing the production of oxidizers such as oxygen-free radical species (ORS). Gentamycin is an antibiotic that has been used to treat several types of bacterial infections and was reported to promote hepatic damage through raising aspartate transaminase and alanine aminotransferase enzymes in addition to lowering the plasma albumin level. It is demonstrated that garlic protects against gentamycin- as well as acetaminophen-induced hepatotoxicity by improving antioxidant status, and regulating oxidative stress [50]. As ROS seems to be at the core of many ailments, it is justified to assume that the antioxidant effect of garlic might be through modulation of ROS, increasing glutathione and cellular antioxidant enzymes [53]. Moreover, garlic extract was found to increase the activities of some antioxidant enzymes (e.g., superoxide dismutase (SOD)) and decrease glutathione peroxidase (GSH-Px) in hepatic tissues of rats. Notably, several reports indicated that AGE rich in flavonoid, phenol, and different sulfur compounds e.g., SAC shows high radical scavenging activity [65]. Additionally, AGE acted by stimulating the expression of different antioxidant enzymes, namely glutamate-cysteine ligase modifier (GCLM) and heme oxygenase-1 (HO-1) subunit by the nuclear factor erythrobia-2 related factor 2 (Nrf2)-antioxidant response element (ARE) pathway that is responsible for human endothelial cells protection against oxidative stress [66]. Alliin, the major compound isolated from AGE, showing wide-spectrum antioxidant activities by controlling ROS generation and preventing mitogen-activated protein kinase (MAPK). Moreover, it was reported to prevent ROS production by inhibiting NADPH oxidase 1, and thus, inhibiting the osteoclast fusion caused by receptor activator of nuclear factor-kappa B ligand (RANKL) [67]. Allicin, DADS, and DATS are the main antioxidative compounds that showed an antioxidant effect in lower doses at the physiological level [54]. Saponins extracted from garlic were reported to scavenge intracellular ROS and protect mouse-derived C2C12 myoblasts towards growth inhibition and H_2_O_2_-induced DNA damage [68]. Interestingly, Abdel-Daim et al. [69] reported that DAS exhibited potent antioxidant and cytoprotective activities and these activities may be due to suppressing the enzymatic activity of cytochrome P450-2E1 and thereby reducing the generation of reactive oxygen and nitrogen species or by inducing the mRNA expression of Nrf2 and heme-oxygenase 1 enzyme.

#### 4.3.2. Anti-Inflammatory Activity

Garlic extracts and its related phytochemicals have been reported to possess anti-inflammatory activity. A study reported that the garlic extracts remarkably impaired the liver inflammation and damage caused by *Eimeria papillate* infections [70]. Hobauer et al. [71], as well as Gu et al. [72], observed that the anti-inflammatory activity of garlic is caused by inhibiting the emigration of neutrophilic granulocytes into epithelia. Aged black garlic (ABG) exhibited potent antioxidant activities and these activities may be responsible for its anti-inflammatory activity. The ABG chloroform extract acts by reducing NF-κB activation in human umbilical vein endothelial cells caused by tumor necrosis factor-α (TNF-α). Moreover, ABG methanolic extract was reported to prevent the cyclooxygenase-2 (COX-2) and prostaglandin E_2_ (PGE_2_) production by NF-κB inactivation [73]. You et al. [74] investigated the anti-inflammatory effect of ABG and they reported that this activity may be attributed to the direct suppression of toll-like receptor 4 (TLR4) signaling cascade activation in macrophages, reducing nuclear NF-κB level and improving the NF-κB and IκB cytosolic levels in LPS-activated RAW264.7 cells. Additionally, they revealed that ABG extract may act by another mechanism of action by inhibiting the iNOS and COX-2 expression, and thus, prevented the NO, interleukin-6 (IL-6) and TNF-α formation of in LPS-activated RAW264.7 cells and TPA-mediated dermatitis in mice. Allicin demonstrated a defensive mechanism against pathogens by its ability to enhance the activity of immune cells and influence signaling pathways associated with these immune cells. Moreover, allicin works on T-cell lymphocytes by inhibiting the SDF1α chemokine which is associated with the weakness of the dynamic structure of the actin cytoskeleton [75], in addition to this, it leads to inhibit the Transendothelial migration of neutrophils. Notably, Abdel-Daim et al. [76] reported that the anti-inflammatory activity of DAS induced by diminishing the expression of the inflammatory cytokines (e.g., NF- κB, IL-1β, and TNF-α), and the ROS generation by suppressing CYP-2E1 hepatic enzyme. Another report indicated that thiacremonone (a sulfur compound isolated from garlic) prevents neuroinflammation and amyloidogenesis by blocking the NF-κB activity, and therefore can be used to treat neurodegenerative disorders (e.g., Alzheimer’s disease) related to inflammation [77]. 

### 4.4. Anticancer Activity

Raw garlic extract was found to be the most effective and highly specific anticancer drug when compared with 33 raw vegetable extracts against different cancer cells without affecting the non-cancerous cells [78]. Shang et al. [68] reported that the anticancer mechanisms of garlic extracts were attributed to the inhibition of cell growth and proliferation, regulation of carcinogen metabolism, stimulation of apoptosis, prevention of angiogenesis, invasion, and migration and thus reducing the anticancer agent’s negative effects. Interestingly, in 1960, tumor cells were reported to be killed when incubated in an allicin solution [63]. Allicin isolated from garlic was reported to suppress colorectal cancer metastasis through enhancing the immune function and preventing the formation of tumor vessels as well as survivin gene expression to enhance the cancer cell’s apoptosis. It also can enhance the treatment of pancreatic cancer thereby invert gene silencing and restrain cancer cell proliferation [79]. Furthermore, Zhang et al. [80] revealed that allicin can prevent gastrointestinal cancer cells MGC 803 proliferation and induce apoptosis, which can be accomplished through enhancing p38 expression and cleaved caspase 3. Allicin-derived polysulfanes have been reported to target microtubules, which lead to interruption of the cell-cycle and finally to apoptosis. Several studies reported the activity of allicin in preventing cell proliferation [81] by targeting tubulin that shapes the mitotic spindle and thus inhibits cell division [82]. Iciek et al. [83] have reported the anti-tumor properties of organo-sulfur compounds (OSC) including allicin, DADS, alliin, DAS, allyl mercaptan (AM), and S-allyl cysteine (SAC), isolated from garlic. In addition, garlic powders inhibited the DNA damage caused by N-nitrosodimethylamine in the liver when administered to rats by 35% and this effect was due to the high concentration of alliin up to 60% in the samples [84]. Notably, Fleischauer and Arab [85] reported that continuous garlic intake could decrease different kinds of cancer propagation such as lung, colon, stomach, breast, and prostate. Piscitelli et al. [86] reported that garlic reduced the plasma concentrations of saquinavir by about 50% in healthy participants, after 3-week of a garlic supplement uptake, in addition to this, many researchers evaluated the antitumor and cytotoxic actions of garlic and its related constituents in vitro and in vivo. Moreover, *Z*-ajoene has shown anti-proliferative activities against different types of cancers and it inhibits the growth of human breast cancer cells and glioblastoma multiforme cancer stem cells (GBM CSC) [68]. It was found to stimulate apoptosis in human leukemic cells by promoting the peroxide production, caspase-3-like and caspase-8 activities [87].

### 4.5. Anti-Alzheimer’s Disease Activity

Alzheimer’s disease (AD) is the main cause of dementia in the elderly with neurodegenerative and cerebrovascular disorders [88]. Acetylcholinesterase (AChE) is the main enzyme that converts the acetylcholine (ACh) in the nervous system to acetate and choline [89]. ACh depletion in the central nervous system has been involved in the pathophysiology noticed in AD [90], therefore, donepezil (AChE inhibitor) was effective in the management/prevention of AD. Surprisingly, oil from garlic bulbs suppressed AChE activity of cerebral cortex synaptosome and exhibits antioxidant properties, thus, inhibiting AChE activity in vitro [91] as well as their ability to scavenge diphenyl-1-picrylhydrazyl (DPPH) free radical that are used to evaluate the compound’s ability to act as hydrogen donors or free radical scavengers and to assess the antioxidant activity of food [92] and reduce Fe^3+^ to Fe^2+^ could be suggested as the possible mechanism of action for their neuroprotective potential [86]. 

Noteworthy, the inclusion of garlic in cholesterol-fed rats’ diet remarkably reduced the total glycosaminoglycans (GAGs) concentration in heart and aorta. This may be due to the enhanced GAGs degrading enzyme activity such as hyaluronidase, *β*-N-acetylhexosaminidase arylsulfatase and *β*-glucuronidase [20]. Sulfated GAGs are involved in lipid aggregation in the lesion development due to their ability to bind to plasma lipoproteins, mainly LDL. Moreover, sulfated GAGs stimulated the neurotoxic activities of various amyloidogenic peptides such as A in AD [20]. Borek [93] evaluated the neuroprotective effect of AGE using an animal model and they showed that AGE protected the brain from neurodegenerative diseases by preventing brain injury following ischemia, saving neurons toward apoptosis, and inhibiting oxidative death caused by β-amyloid [94]. Moreover, Mbyirukira and Gwebu [95] reported that AGE or SAC inhibits the brain’s frontal lobe degeneration, promotes memory and learning retention, and prolongs the lifespan. 

Based on the amyloid hypothesis, aggregated β-amyloid (Aβ) accumulation in the brain is believed to be the pathological factors that drive the onset of AD. It has been suggested that the formation of the neurofibrillary tangles contain τ-protein and synaptic degradation caused by the imbalance consequences between Aβ clearance and Aβ production. Haider et al. [96] reported that the prolonged garlic uptake is related to promoting the memory function by affecting the levels of the neurotransmitter, serotonin. The in vivo consumption of *A. sativum* extracts have shown that it improves memory by eliminating free radicals that cause oxidative damage and inhibit AChE enzyme [97]. It was noted that allicin inhibits AChE and butyrylcholinesterase (BuChE) enzymes (enzymes that break down neurotransmitter choline) which successively increased ACh concentration in the brain. Thus, delayed cognitive decline and dementia [98]. 

Garlic is also investigated to have immunomodulatory, anti-inflammatory, and antioxidant effects and this focused on the question of whether the known effect of processed garlic and its related compounds mainly allicin in inhibiting AChE and BuChE enzymes [29]. Combination therapy of allicin with cholinesterase inhibitors (ChEIs) including; rivastigmine, galantamine, and donepezil are now the most commonly used for the treatment of AD [99] as they have the ability to correct the cholinergic deficiency seen with AD. Antioxidants such as tocopherol, selegiline, and ascorbic acid (vitamin C) were examined as a possible preventive therapy for AD, and they show delayed functional deterioration in AD patients [100]. Anti-inflammatory drugs such as NSAIDs have been used as a potential treatment in AD because of their capacity to bind to and stimulate the nuclear receptor peroxisome proliferator-activated receptor (PPAR)-γ as well as their direct effects on the amyloid formation [101].

It should be noted that AChE inhibitors could be part of any combination therapy against AD [100]. For instance, Millard et al. [102] reported that AChE incubated with allicin produced rapid inactivation that was concentration and time-dependent. Many results showed concentration-dependent inhibition of bovine AChE by allicin complementing the previous finding. However, different cholinesterase inhibitors such as donepezil, rivastigmine, and tacrine are used to treat AD, and their side effects are becoming increasingly remarkable [103,104]. Therefore, the search for new derivatives extracted from the natural product with a dual function and lower side effects could be useful for patients with AD.

Allicin is a small lipophilic molecule that can suppress BuChE and AChE, and therefore, enhances ACh concentration, which is decreased remarkably in AD patient’s brains [103]. Recently, allicin has been shown to have a protective effect on ischemic or traumatic neuronal damage controlled by apoptosis and oxidative stress pathways [105]. 

### 4.6. Activities Related to Metabolic Diseases

#### 4.6.1. Effect on Dyslipidemia

Dyslipidemia is known to be the main cause of myocardial infarction and cardiovascular diseases and it is defined by high levels of triglyceride (TG), LDL, total cholesterol (TC), and low HDL level [106]. Interestingly, various evidence encourages the significant and crucial role of garlic preparations and its phytochemicals in treating hypercholesterolemia by preventing the cholesterol biosynthesis in the liver as well as inhibiting low-density lipoproteins (LDL and HDL) oxidation. Moreover, garlic reduces the cholesterol level either by stimulating the acidic and neutral steroids excretion or by reducing the cholesterogenic and lipogenic effects of fatty acid synthase, 3-hydroxy-3-methyl-glutaryl-CoA reductase, malic, and glucose-6 phosphate dehydrogenase in hepatocytes [107]. Garlic was found to have an important effect on dyslipidemia by significantly decreased serum TC, TG, and LDL levels and moderately elevated HDL cholesterol [108]. Various experimental and clinical trials were performed in animals and humans using various garlic preparations and they exhibited disputable results. They claimed that these variable results were attributed to the differences in garlic preparation composition, amount of active sulfur compounds exist in each preparation and the mechanism by which they act. For instance, Iweala et al. [108] reported that ethanolic garlic extract uptake to albino rabbits resulting in decreased their cholesterol level and body weight. Campbell et al. [109] reported that AGE significantly prevented the development of thickened, lipid-filled lesions in the preformed neointima generated from balloon-catheter harm of the right carotid artery in rabbits fed with cholesterol. In clinical trials in patients, Sobenin et al. [110,111] revealed that garlic administration at a dose of 300 and 60 mg/day for 12 months and 12 weeks, respectively decreased TC, TG, and LDL while increased HDL. Moreover, Ashraf et al. [112] garlic tablets administration at a dose of 600 mg/day for 12 weeks in diabetic patients with dyslipidemia results in high HDL and low LDL and TC levels.

#### 4.6.2. Effect on Diabetes Mellitus

Ethanolic garlic extracts exhibited an antidiabetic effect against streptozotocin- and alloxan-induced diabetic mice and rabbits by activating the insulin secretion from parietal cells of the pancreas [113]. Another clinical study examined the antidiabetic effect of garlic pills administration at 900 mg/day in patients with type II diabetes and hyperlipidemia and they reported that garlic pills decrease the cholesterol, serum lipids, and fasting blood sugar [114]. Moreover, allyl propyl disulfide, allicin, cysteine sulfoxide, and S-allyl cysteine sulfoxide decreased the blood glucose level by preventing the insulin activation caused by liver, enhancing the secretion of insulin from pancreatic beta cells, isolation of insulin from the bonded forms, and increasing the cell sensitivity to insulin [114]. Zhai et al. [115] reported that the activity of alliin in reducing diabetes mellitus in rats was similar to that demonstrated by glibenclamide and insulin. Garlic oil also was reported to decrease the serum amylase, serum aspartate and alanine transferases, and serum alkaline and acidic phosphatase in diabetic rats.

#### 4.6.3. Effect on Obesity

Obesity is the most common health problems that may lead to many ailments like hypertension, dyslipidemia, cardiovascular disorders, and metabolic syndrome. Garlic extracts have been reported for their activity in reducing body weight, adipose tissue mass and improved plasma lipid profiles in mice with high-fat diet-induced obesity and these effects mediated by the downregulation of multiple genes expression that is included in adipogenesis along with upregulation of the mitochondrial inner membrane proteins expression [116]. Moreover, Lee et al. [116] revealed that the antiobesity effect of garlic extracts attributed to stimulation of AMP-activated protein kinase (AMPK) as well as increased thermogenesis and decreased multiple genes expression that is included in adipogenesis. Ajoene isolated from garlic extracts was found to stimulate apoptosis, decrease the fat accumulation in 3T3-L1 adipocytes and dramatically decrease the body weight gain in mice without affecting the amount of food intake [117]. 1,2-vinyldithiin also has been reported to prevent the human preadipocytes differentiation and decrease lipid accumulation by decreasing the C/EBP*α*, PPAR*γ*2, and LPL expression and the PPARγ effect in human adipocytes [118]. 

#### 4.6.4. Antihypertensive Activity

Varshney and Budoff [119] reported the essential function of garlic in the control of cardiovascular risk factors as it is known to significantly decrease systolic as well as diastolic blood pressure. Garlic formulations have been broadly used to inhibit and relieve cardiovascular disorders such as hypertension, arrhythmia, thrombosis, hyperlipidemia, and atherosclerosis [19,120]. Several experimental and human studies reported the antihypertensive effect of garlic extracts and its derived bioactive molecules. For example, Sobenin et al. [121] showed the plasma fibrinolytic activity of garlic extracts and they found that it increased fibrinolytic activity in both healthy and acute myocardial infarction participants. Moreover, in vivo experiment exhibited the antihypertensive effect of aqueous garlic extract in ‘2 kidney 1-clip’ model of hypertension in rat by reducing thromboxane B2 and prostaglandin E2 level and thereby reduced hypertension in tested rats [122]. Garlic administration at a dose of 100 mg/kg for 5 days resulted in complete prevention of acute hypoxic pulmonary vasoconstriction caused by endothelin-1 in isolated rat pulmonary arteries and they found that garlic acts by reducing endothelin 1 and angiotensin II production [120]. The mechanism of antihypertensive effect of garlic extracts is that garlic contains many active sulfur molecules that have been shown to stimulate endothelium-constricting and -relaxing factors leading to lower blood pressure. Garlic has also been shown to stimulate the production of both nitric oxide (NO) and hydrogen sulphide (H_2_S) that finally leads to vasodilation. Therefore, garlic is used as a medicinal plant for controlling blood pressure worldwide [123]. Furthermore, garlic exhibited a significant role in inhibiting thrombosis as well as platelet adhesion or aggregation in humans. The AGE was reported to prevent both ADP-activated platelets binding to immobilized fibrinogen and platelet aggregation by inhibiting GPIIb/IIIa receptor and increasing cAMP [124]. Furthermore, garlic has been reported to reduce the risk of plasma viscosity, unstable angina, and peripheral arterial occlusive disorders and increase the elasticity of the blood vessels and perfusion of capillaries [87]. The gamma-glutamylcysteine isolated from garlic was reported to decrease the blood pressure by inhibiting the angiotensin-converting enzyme (ACE). Dubey et al. [122] revealed that allicin shows remarkable activity in reversing systolic blood pressure caused by dexamethasone and enhances body weight and food intake in hypertension caused by dexamethasone in rats. 

**Figure 1:**
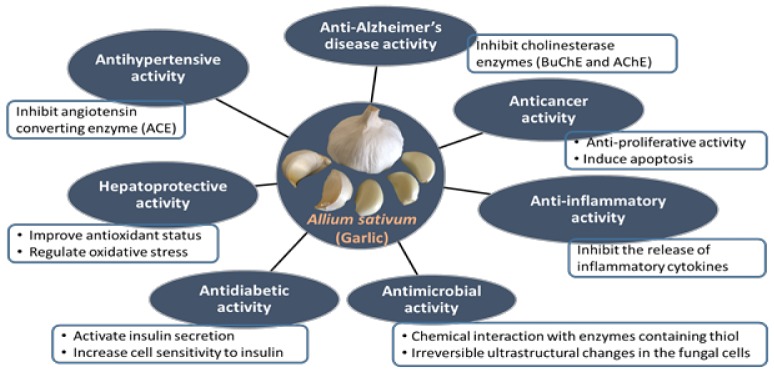
Schematic representation of different pharmacological activities of garlic (*Allium sativum*) and their mechanisms.

Few pharmacological effects of garlic and its related bioactive compounds are shown in Table 2. Some of the mechanisms of action related to these activities are shown in Figure 1.

### 4.7. Recommended Dose and Toxic Side Effects of Garlic

#### 4.7.1. Recommended Dose 

The generally recommended doses of the daily garlic uptake for the elderly are 4 g of raw garlic or 7.2 g of AGE or one dried garlic powder tablet twice to thrice per day [125]. Rana et al. [82] revealed that oral or intraperitoneal administration 50 mg/kg of garlic to rats did not show any effect on liver and lung tissue, while intake garlic at 250, 500, and 1000 mg/kg per day led to acute deformities in the rat’s liver and lung tissue, suggesting the dose-related toxicity. While garlic intake at a dose of 500 and 1000 mg/kg/day remarkably decreased the auto-antioxidants without changing the lipid peroxidation level, whereas the daily intake of 1000 mg/kg resulted in morphological deformities in the liver under light microscopy and ultrastructural levels. Moreover, histological examination revealed nonspecific focal injury to the hepatocytes. In addition to this, Mikaili et al. [51] reported that garlic bulb extracts ingestion to male and female rats at 300 and 600 mg for 21 days, led to delayed growth and affects the biological and histological parameters. In particular, Asdaq and Inamdar. [126] indicated that the combination therapy of 250 mg/kg of garlic with hydrochlorothiazide shows synergistic antihypertensive and cardioprotective activities against toxicity caused by fructose and isoproterenol. While the combination therapy of 250 mg/kg of garlic with propranolol revealed a remarkable elevation in the antioxidant enzymes activities throughout ischemic injury [33]. 

#### 4.7.2. Adverse Effects and Toxicity

Although the US Food and Drug Administration (FDA) considers garlic safe for humans, it can induce gastric agitation especially if ingested in high doses by sensitive people. To assess the safety of garlic, randomized controlled trials were performed, side effects such as insomnia, vomiting, heartburn, dizziness, diarrhea, tachycardia, nausea, bloating, flushing, headache, mild orthostatic hypotension, sweating, offensive body odor, and flatulence were observed [82]. Ingestion of raw garlic in high doses on an empty stomach can induce changes in the intestinal flora, flatulence and gastrointestinal upset [127]. Moreover, blisters dermatitis and burns were observed from raw garlic local applications [127]. Garlic does not seem to affect the drug metabolism, although recent reports on healthy participants show inconsistent results regarding the garlic effect in the pharmacokinetics of protease inhibitors, as well as anticoagulants due to its antithrombotic properties [46]. Many surgeons recommended stopping garlic administration in high doses up to 7 to 10 days prior to operation due to its effect to prolong the bleeding time that was observed in one patient with epidural spontaneous hematoma [46]. 

Previous in vivo experiments revealed that prolonged feeding of raw garlic in high doses led to weight loss and anemia due to red blood cells (RBCs) lysis, while administration of 5 mL/kg of raw garlic juice resulted in stomach injury that led finally to death [20]. Additionally, the chronic administration of 50 mg garlic powder per day led to anti-androgenic effects by inhibiting spermatogenesis in rats, leading to decrease sialic acid concentration in the seminal vesicles, testes, and epididymis with reduced Leydig cell function [82]. Oxidative hemolysis is the main toxicological mechanism of *Allium*-derived sulfur compounds and it is distinguished by methemoglobinemia development and Heinz body formation in the RBCs [128]. Initially, several clinical symptoms were observed including depression, vomiting, loss of appetite, abdominal pain, diarrhea, as well as anemia associated with pale mucous membrane, jaundice, rapid heart and respiratory rates, weakness, and hemoglobinuria [128]. *Allium* poisoning symptoms may appear after one day or several days of its ingestion based on the amounts taken [129]. 

Previous reports have reported the cardiovascular effects of garlic including potentially irreversible antiplatelet activity, anticoagulant, fibrinolytic activity, a remarkable decrease in platelet accumulation and mixed activity on fibrinolytic effectiveness [130]. Chen et al. [131] revealed that dehydrated raw garlic powder when administered orally resulted in acute injury to the gastric mucosa, whilst Yuncu et al. [132] reported that AGE, the sulfur-free compound, protects the intestinal mucosa of experimental animals. Clinical studies reported that low doses of garlic are safe, whereas therapeutic doses might cause mild gastrointestinal disorders, while high doses have been reported to cause liver damage [82,123,133].

Allicin is a membrane-permeable compound that can enter cells easily and interact with cellular thiols such as glutathione or cysteine residues in proteins [37,89,90] as well as enzymes containing reactive cysteine and this may be the potential interpretation of allicin’s toxicity [63]. Interestingly, Rana et al. [82] revealed that garlic powder or allicin at a concentration of 200 mg/mL can cause significant cell damages in the isolated rat liver.

## 5. Combination Therapy with Other Drugs 

Recently, Mohammadi et al. [134] revealed the potent activity of garlic and ezetimibe combined treatment in reducing plasma LDL-C and TC, and thus, inhibiting the absorption of intestinal cholesterol and reducing the cardiovascular disorders risk factors. Asdaq and Inamdar [33] reported the combined effect of garlic homogenate and propanol in attenuating the isopropanol-mediated cardiac *β*_1_-receptors excessive stimulation, myocardial hypoperfusion, electrolyte imbalance, glycogen depletion, free radical injury, thermogenesis, lipid peroxidation, lipid accumulation, and electrocardiographic disturbances. They indicated that garlic homogenate is a good combination therapy as it reduces the dose and toxic side effects of propanol, which may assist in decreasing repeated higher doses of propanol. Mikaili et al. [51] reported the combined effect of allicin with polymyxin B against various yeasts and filamentous fungi and this combination therapy was found to increase the permeability of plasma membrane in *Saccharo cerevisiae*. Moreover, the combination treatments of garlic with captopril showed a higher synergistic effect regarding ACE inhibition [135]. Notably, the combination treatment of AGE with methotrexate showed improved activity against the significant increase in liver function enzymes, proinflammatory cytokines and antioxidants [136]. Recent researches reported that the fresh garlic extracts and antibiotics combination therapy resulted in high antibacterial activity. For instance, Ismail et al. [137] revealed that aqueous garlic extract-ampicillin combined treatment exhibited a potent synergetic effect towards *Kl. pneumoniae*, *Sal. typhi*, *E. coli*, and *P. aeruginosa*. Moreover, Vathsala and Murthy [138] revealed the potent immunomodulatory and anti-plasmodial effect of garlic–artemether combination treatment. They reported that this combined therapy may have a potential role in reducing organ injury and protecting against *Plasmodium* species by affecting NO production, suggesting novel treatment options against malaria [138,139].

## 6. Conclusions

This review focused on the chemical constituents and pharmacological activities of *A. sativum*. Sulfur-containing compounds such as alliin, allicin, ajoenes, vinyldithiins, and sulfides, are the main constituents isolated from *A. sativum* extracts. Extracts and isolated compounds from *A. sativum* reported to possess several biological properties including anticarcinogenic, antioxidant, antidiabetic, renoprotective, anti-atherosclerotic, antibacterial, antifungal, antiprotozoal, and antihypertensive activities. Garlic is also well-known to have immunomodulatory and anti-inflammatory activities. Allicin, the active substance of the garlic, can induce gastric agitation especially if administered in high doses. In addition to that, *A. sativum* has been reported to affect the pharmacokinetics of antiretroviral drugs, as well as anticoagulants. Thus, proper consideration should be taken when using garlic as a medicine for the treatment of different diseases.

## Figures and Tables

**Table 1:** List and structures of some of the sulfur-containing compounds isolated from *Allium sativum*.

Compounds	Molecular formula	Structure
**Alliin**	C_6_H_11_NO_3_S	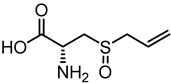
**Allicin**	C_6_H_10_OS_2_	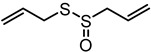
***E*-Ajoene**	C_9_H_14_OS_3_	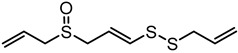
***Z*-Ajoene**	C_9_H_14_OS_3_	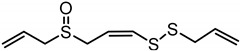
**2-Vinyl-4H-1,3-dithiin**	C_6_H_8_S_2_	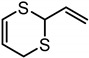
**Diallyl sulfide (DAS)**	C_6_H_10_S	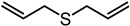
**Diallyl disulfide (DADS)**	C_6_H_10_S_2_	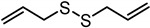
**Diallyl trisulfide (DATS)**	C_6_H_10_S_3_	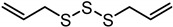
**Allyl methyl sulfide (AMS)**	C_4_H_8_S	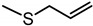

**Table 2:** The pharmacological activity of garlic (*Allium sativum*) and its related compounds.

Activities	Bioactive Compound	Mechanism of Action	References
Antibacterial	Allicin	Chemical interaction with enzymes containing thiol	[54]
Antifungal	DADS	Irreversible ultrastructural changes in the fungal cells, loss of structural integrity and affected the germination ability	[44]
	DATS		
Antiviral	Allicin	Chemical interaction with enzymes containing thiol	[58]
	DATS	Enhancing Natural killer-cell (NK-cell) activity that destroys virus-infected cells	
Antiprotozoal	Allicin	Preventing the parasite’s RNA, DNA and protein synthesis.	[58]
	DATS		
	Ajoene	Inhibiting the human glutathione reductase and *T. cruzi* trypanothione reductase	[61]
Antioxidant	Allicin, DADS, and DATS	Modulation of ROS, increasing glutathione and cellular antioxidant enzymes	[54]
	Alliin	Controlling ROS generation and preventing mitogen-activated protein kinase (MAPK)	[67]
	DAS	Suppressing the enzymatic activity of cytochrome P450-2E1, reducing the generation of reactive oxygen and nitrogen species	[69]
Anti-inflammatory	Allicin	Enhancing the immune cell activity f, inhibiting the SDF1α chemokine and Transendothelial migration of neutrophils	[60]
	DAS	Diminishing the expression of the inflammatory cytokines (e.g., NF- κB, IL-1β, and TNF-α), and ROS generation by suppressing CYP-2E1 hepatic enzyme	[76]
	Thiacremonone	Blocking the NF-κB activity	[77]
Anti-cancer	Allicin, alliin, DADS, DAS	Enhancing p38 expression and cleaved caspase 3.	[80]
	*Z*-Ajoene	Stimulating apoptosis in human leukemic cells, promoting the peroxide production, caspase-3-like, and caspase-8 activities	[87]
Immunomodulatory	Allicin	Suppressing BuChE and AChE	[105]
Anti-obesity	Ajoene	Decreasing the fat accumulation in 3T3-L1 adipocytes and dramatically decreases the body weight gain	[117]
	1,2-Vinyldithiin	Decreasing the C/EBP*α*, PPAR*γ*2, and LPL expression and the PPAR*γ* effect in human adipocytes	[118]
Antidiabetic	Allyl propyl disulfide, allicin, cysteine sulfoxide, and S-allyl cysteine sulfoxide, alliin	Decreasing the insulin secretion from pancreatic cells, increasing liver metabolism, and thus enhancing the short-acting insulin production	[114,115]
Hypolipidemic, hypocholesterolaemic	Different garlic preparations	Decreasing serum TC, TG, and LDL levels and moderately elevating HDL cholesterol	[107]
Anti-Atherosclerotic, antithrombotic	Different garlic preparations	Preventing ADP-activated platelets binding to immobilized fibrinogen and platelet aggregation, inhibiting GPIIb/IIIa receptor and increasing cAMP	[120]
Antihypertensive	Gamma-glutamylcysteine	Inhibiting the angiotensin-converting enzyme	[87]

## Abbreviations

AGE	aged garlic extract
PCSO	S-propyl cysteine-sulfoxide
MCSO	S-methyl cysteine-sulfoxide
NAC	N-Acetylcysteine
SAC	S-Allyl-cysteine
SAMC	S-ally-mercapto cysteine
PLP	pyridoxal phosphate
DAS	Diallyl sulfide
DADS	Diallyl disulfide
DATS	Diallyl trisulfide
AMS	Allyl methyl sulfide
AMDS	allyl methyl disulfide
SGF	simulated gastric fluid
SIF	stimulated intestinal fluid
HCMV	Human *Cytomegalovirus*
NK-cell	Natural killer-cell
ORS	oxygen-free radical species
SOD	superoxide dismutase
GSH-Px	glutathione peroxidase
GCLM	glutamate-cysteine ligase modifier
HO-1	heme oxygenase-1 subunit
Nrf2	nuclear factor erythrobia-2 related factor 2
ARE	antioxidant response element
MAPK	mitogen-activated protein kinase
RANKL	receptor activator of nuclear factor-kappa B ligand
ABG	Aged black garlic
TNF-α	tumor necrosis factor-α
COX-2	cyclooxygenase-2
PGE_2_	prostaglandin E_2_
TLR4	toll-like receptor 4
IL-6	interleukin-6
GBM CSC	Glioblastoma multiforme cancer stem cells
AChE	Acetylcholinesterase
Ach	acetylcholine
AD	Alzheimer’s disease
BuChE	butyrylcholinesterase
TG	triglyceride
TC	total cholesterol
UCP	mitochondrial inner membrane proteins
AMPK	AMP-activated protein kinase
NO	nitric oxide; H_2_S: hydrogen sulphide
FDA	Food and Drug Administration
RBCs	red blood cells
ACE	Angiotensin-converting enzyme
IUPAC	International Union of Pure and Applied Chemistry
